# AlfaPang: alignment free algorithm for pangenome graph construction

**DOI:** 10.1186/s13015-025-00277-7

**Published:** 2025-05-15

**Authors:** Adam Cicherski, Anna Lisiecka, Norbert Dojer

**Affiliations:** Institute of Informatics, University of Warsaw, Banacha 2, 02-097 Warsaw, Poland

**Keywords:** Pangenome, Variation graph, Genome alignment, Population genomics

## Abstract

The success of pangenome-based approaches to genomics analysis depends largely on the existence of efficient methods for constructing pangenome graphs that are applicable to large genome collections. In the current paper we present AlfaPang, a new pangenome graph building algorithm. AlfaPang is based on a novel alignment-free approach that allows to construct pangenome graphs using significantly less computational resources than state-of-the-art tools. The code of AlfaPang is freely available at https://github.com/AdamCicherski/AlfaPang.

## Background

Pangenome (or variation) graphs serve as models for joint representation of populations of genomes [1–5]. They have proven to be useful in analyzing sequence evolution and variation [6, 7], as well as in reducing the so-called reference bias in the analysis of experimental data [8, 9].

A first draft of the human pangenome reference, constructed from 47 high-quality assemblies, was recently published by the Human Pangenome Reference Consortium, demonstrating its effectiveness in reducing errors in the detection of small and structural variants [10]. Additionally, the use of a pangenome has improved short-read mapping, as well as ChIP-seq and ATAC-seq analyses. To further enhance its accuracy and completeness, the consortium plans to expand the reference to 350 assemblies in the coming years.

However, the success of the pangenome-based approaches depends on the existence of efficient construction methods, applicable to large collections of genomes. Most pangenome building algorithms adapt the approaches used in whole genome alignment tools. Early versions of the VG toolkit [8] constructed pangenome graphs iteratively, i.e. aligning consecutive sequences to a current graph. In the current version of VG, by default, graphs are constructed from genomic sequences using Minigraph-Cactus [11], which aligns all genomes to a reference genome.

In both approaches the outcome depends on an arbitrary choice of genome order (VG) or the reference (Minigraph-Cactus). To avoid such biases, several alternatives have recently been proposed. seqwish [12] builds pangenome graphs from all-to-all pairwise genome alignments. Unfortunately, the construction doesn’t scale linearly with respect to the number of genomes, and the final graph requires refinement. The last problem was addressed in pggb [13] – a pipeline that builds a pangenome graph in three steps:

1. All-to-all genome alignment (wfmash),
2. Graph inference from pairwise alignment (seqwish),
3. Graph refinement (smoothxg+gfaffix).

All the above-mentioned tools build variation graphs, in which the concatenation of the labels of all the nodes in a path constitutes the sequence represented by the path. As a result, variation graphs provide an intuitive, common coordinate system in which each base pair in any genomic sequence is uniquely represented in the graph. One of the most important alternatives are de Bruijn graphs, in which nodes are uniquely labeled with *k*-mers, and edges connect nodes with labels that are consecutive *k*-mers in the represented sequences (and thus overlap with $k-1$ characters). Consequently, a single base pair occurs in *k* different node labels (i.e. the *k*-mers covering the base pair), and therefore de Bruijn graphs pose a challenge for downstream analysis, especially in terms of annotation, visualization and information extraction [14]. On the other hand, their structure is strictly determined by the parameter *k*, and hence the order and reference bias problems do not occur in building de Bruijn graphs. Moreover, the construction is conceptually simple, and optimized building algorithms such as TwoPaCo [15] or bifrost [16] are orders of magnitude faster than alignment-based building algorithms for variation graphs.

A bridge between both models that could combine their advantages was proposed in [17]. The paper introduces the notion of a string graph, which is a generalization of both variation graph and de Bruijn graph. Moreover, the authors propose an axiomatization of the desired properties of representing a sequence collection in such a graph. It is shown that the axioms are always satisfied in de Bruijn graphs and that they determine the structure of variation graphs up to merging unbranched paths into single nodes and the opposite operation. Furthermore, authors explore the relationship between de Bruijn graphs and variation graphs satisfying the axioms to design an algorithm transforming the former into the latter. The proposed transformation algorithm can potentially be used as a crucial component of an efficient variation graph building pipeline.

In this paper we go one step further to achieve a more efficient construction of the variation graph. We design and implement AlfaPang – an algorithm that builds directly from the input sequences a variation graph satisfying the axioms introduced in [17]. We show that replacing the first two steps of pggb with our algorithm results in significant efficiency improvement and yields output graphs of similar properties.

The rest of the paper is organized as follows. In section "Representing sequences with variation graphs" we introduce the necessary notation and prove theoretical results underlying the correctness of our algorithm. In section "AlfaPang algorithm" we describe the algorithm and study its complexity. Sections "Design of experiments" and "Results" present experiments’ design and results, respectively.

## Representing sequences with variation graphs

In this section we give a mathematical formalism behind our algorithm. We start with introducing directed variation graphs, i.e. directed graphs with nodes labeled with sequences. We provide some necessary definitions, including the extension of the labeling function to paths, the quotient graph construction, and the notion of singular (i.e. single-character-labeled) graphs. Because every variation graph can be transformed to an equivalent (in the sense defined in [17]) singular one, in the rest of this section we restrict our attention to singular graphs.

In subsection "Representations of collections of sequences" we formalize the notion of a singular directed variation graph representing given set of sequences. Then we introduce two properties of such representations: *k*-completeness and *k*-faithfulness. Intuitively, the *k*-completeness states that every occurrence in the sequences of the same *k*-mer is represented by the same path in the graph, while *k*-faithfulness states that different sequence fragments can be represented by the same path only when it is essential to satisfy *k*-completeness. We conclude subsection "Representations of collections of sequences" showing that, given the set of sequences, these properties uniquely define the structure of their representation by a singular directed variation graph.

In the last subsection, we introduce bidirected variation graphs and show how the above concepts and results generalize to them. In bidirected variation graphs paths pass through nodes in one of two possible directions, corresponding to two DNA strands. In this way they naturally represent the double-stranded structure of DNA, which helps modeling the collections of genomes with sequence differences resulting from inversions. The directed case is described first for clarity, since the bidirected case is technically more complex but conceptually similar.

### Directed variation graphs

A *directed variation graph* is a tuple $G=\langle V, E, l \rangle$, where:

- *V* is a set of vertices,
- $E\subseteq V^2$ is a set of directed edges,
- $l: V \rightarrow \Sigma ^+$ is a function labeling vertices with non-empty strings over the DNA alphabet $\Sigma = \{A,C,G,T\}$.

A *path* in a variation graph is a sequence of vertices $\langle v_1,\ldots ,v_m\rangle$ such that $\langle v_{j}, v_{j+1}\rangle \in E$ for every $j\in \{1,\ldots ,m-1\}$. The set of all paths in *G* will be denoted by ${\mathcal {P}}(G)$. The labeling function *l* extends to ${\hat{l}}:{\mathcal {P}}(G)\rightarrow \Sigma ^+$ defined by formula ${\hat{l}}(\langle v_0,\ldots ,v_m\rangle )=l(v_0)\cdot \ldots \cdot l(v_m)$, i.e. the label of the path is the concatenation of the labels of its consecutive vertices.

Assume that $G=\langle V,E,l \rangle$ is a directed variation graph and $\sim$ is an equivalence relation on the set of *G*-nodes satisfying $v\sim v' \Rightarrow l(v)=l(v')$ for all $v,v'\in V$. Then the quotient graph of *G* by $\sim$ can be defined as $G'=\langle V',E',l' \rangle$, where

- $$V'=V/\sim$$,
- $$E'=\{\langle [v]_{\sim }, [v']_{\sim }\rangle \;|\; \langle v,v'\rangle \in E\}$$,
- $$l'([v]_{\sim })=l(v)$$.

The correctness of the definition of $l'$ (i.e. independence on the choice of $[v]_{\sim }$-representative) is guaranteed by the above assumption on $\sim$. Note that the quotient construction saves the labels of paths, i.e. given a path $p=\langle v_1,\ldots ,v_m\rangle$ in *G*, $p'=\langle [v_1]_{\sim },\ldots ,[v_m]_{\sim }\rangle$ is a path in $G'$ and $\hat{l'}(p')={\hat{l}}(p)$.

Given a path $p=\langle v_1,\ldots ,v_m\rangle$, the *subpath* of *p* is any path $p[j_1..j_2]=\langle v_{j_1},\ldots ,v_{j_2}\rangle$, where $1\le j_1\le j_2\le m$. Similarly, $S[j_1..j_2]$ denotes the substring of a string *S* consisting of the characters from positions $j_1,\ldots , j_2$.

If $|l(v)|=1$ for every vertex *v*, the graph is called *singular*. Every variation graph can be transformed into a singular one by splitting each node *v* into an unbranched path with |*l*(*v*)| nodes, labeled with consecutive *l*(*v*) characters. This transformation can be reversed by contracting the edges on the unbranched paths. Contraction reduces the number of nodes and edges, so the reverse transformation leads to more compact graphs. On the other hand, singular graphs provide a simpler and more convenient representation, because the substrings of the string labeling a path *p* are themselves labels of subpaths of *p* (namely, ${\hat{l}}(p[j_1..j_2])={\hat{l}}(p)[j_1..j_2]$ for all $1\le j_1\le j_2\le |p|$). Since the core part of our algorithm builds a singular graph (compressed in the last step), we define the notions of sequence representation and its properties only for singular graphs. However, all these definitions can be generalized to any variation graphs, see [17] for details.

### Representations of collections of sequences

Given a set of sequences ${\mathcal {S}}=\{S_1,\ldots ,S_n\}$, a singular directed variation graph $G\langle V, E, l \rangle$ and $\pi : {\mathcal {S}}\rightarrow {\mathcal {P}}(G)$, we say that $\langle G, \pi \rangle$ *represents* ${\mathcal {S}}$ iff the following conditions are satisfied:

- $${\hat{l}}(\pi (S_i))=S_i$$ for every $$i\in \{1,\ldots ,n\}$$,
- every vertex in *G* occurs in some path $\pi (S_i)$,
- every edge in *G* joins two consecutive vertices in some path $\pi (S_i)$.

We define the set of *positions* in *S* as $Pos(S)=\{\langle i,j\rangle \;|\; 1\le i\le n \wedge 1\le j\le |S_i|\}$. The set of $\pi$-*occurrences* of a vertex *v* is defined as $Occ_{\pi }(v)=\{\langle i,j\rangle \in Pos(S) \;|\; \pi (S_i)[j]=v\}$. Define the *generic representation* of ${\mathcal {S}}$ as the representation $\langle G_0, \pi _0\rangle$, where $G_0=\langle V_0, E_0, l_0 \rangle$ and:

- $$V_0=Pos(S)$$,
- $$E_0=\{\langle \langle i,j\rangle , \langle i,j+1\rangle \rangle \;|\;1\le i\le n \wedge 1\le j<|S_i|\}$$,
- $$l_0(\langle i,j\rangle )=S_i[j]$$,
- $$\pi _0(S_i)=\langle \langle i,1\rangle , \ldots , \langle i,|S_i|\rangle \rangle$$.

#### Lemma 1

For every singular representation $\langle G, \pi \rangle$ of ${\mathcal {S}}=\{S_1,\ldots ,S_n\}$, there exists an equivalence relation $\sim _{\langle G, \pi \rangle }$ on *Pos*(*S*) such that:

1. *G* is isomorphic to a quotient graph of $G_0$ by $\sim _{\langle G, \pi \rangle }$,
2. $$\pi (S_i)=\langle [\langle i,1\rangle ]_{\sim _{\langle G, \pi \rangle }}, \ldots , [\langle i,|S_i|\rangle ]_{\sim _{\langle G, \pi \rangle }} \rangle$$ for all $$i\in \{1,\ldots ,n\}$$.

#### Proof

The relation $\sim _{\langle G, \pi \rangle }$ is defined as follows:

$$\begin{aligned} \langle i,j\rangle \sim _{\langle G, \pi \rangle }\langle i',j'\rangle \iff \pi (S_{i})[j]=\pi (S_{i'})[j'] \end{aligned}$$

Verifying that both conditions are satisfied is straightforward. $\square$

Let $S_i,S_{i'}$ be two (not necessarily different) sequences from ${\mathcal {S}}$ and assume that they have a common *k*-mer $S_i[p..p+k-1]=S_{i'}[p'..p'+k-1]$. We say that $\pi$ *reflects* this common *k*-mer iff it is represented by the same path in the graph, i.e. $\pi (S_i)[p..p+k-1]=\pi (S_{i'})[p'..p'+k-1]$. We say that $\langle G, \pi \rangle$ represents ${\mathcal {S}}$ *k*-*completely* iff all common *k*-mers in ${\mathcal {S}}$ are reflected by $\pi$.

We say the pair of $\pi$-occurrences $\langle i,j\rangle , \langle i',j'\rangle$ of a vertex *v* is:

- *directly k-extendable* iff these occurrences extend to a common *k*-mer reflected by $\pi$, i.e. $\pi (S_i)[j-m..j+m']=\pi (S_{i'})[j'-m..j'+m']$ for some $m,m'\ge 0$ satisfying $m+m'\ge k-1$,
- *k*-*extendable* if there is a sequence of occurrences of *v* that starts from $\langle i,j\rangle$, ends at $\langle i',j'\rangle$ and each two consecutive occurrences in that sequence are directly *k*-extendable.

We say that $\langle G, \pi \rangle$ represents ${\mathcal {S}}$ *k*-*faithfully* if every pair of occurrences of a vertex is *k*-extendable.

**Fig. 1:**
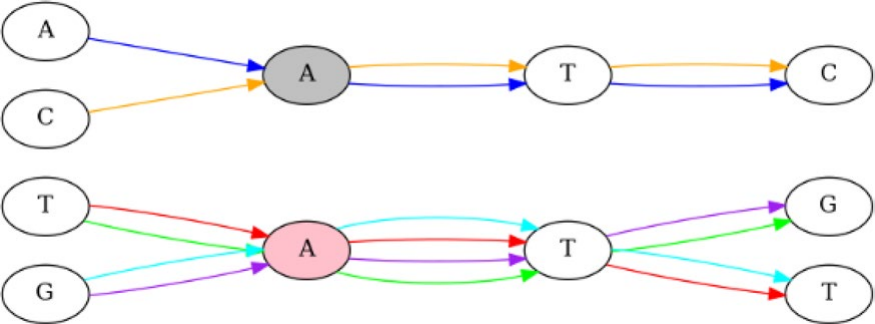
Example of a 3-faithful and 3-complete variation graph. Edge colors are used to mark genomic paths (graph has no multi-edges, they are used only for purpose of paths visualisation). All occurrences of vertex “A” filled with pink are 3-extendable, as occurrences on the red and green paths can both be extended to the path labeled with TAT, on the red and cyan paths to ATT, on the purple and cyan paths to GAT, and on the purple and green paths to ATG. On the other hand, occurrences of the grey vertex “A” on the orange and blue paths can be extended to ATC but are not extendable to any of the previously mentioned 3-mers, and therefore this vertex cannot be merged with the pink one

Note that the *k*-completeness property specifies which fragments of ${\mathcal {S}}$-strings must be unified in the representation, while *k*-faithfulness states that anything that is not a consequence of *k*-completeness cannot be unified (see example on Figure 1).

#### Theorem 1

Let ${\mathcal {S}}=\{S_1,\ldots ,S_n\}$ be a set of sequences. Then a *k*-complete and *k*-faithful representation of ${\mathcal {S}}$ exists and is unique up to isomorphism.

The above theorem is roughly equivalent to Theorems 1 and 2 in [17]. In that paper the proof of the existence was based on the transformation of a de Bruijn graph into a variation graph. Here we propose an alternative proof that leads to a more efficient variation graph construction algorithm.

#### Proof

Let $G_0=\langle V_0, E_0, l_0 \rangle$ be a generic representation of ${\mathcal {S}}$. We define a binary relation $\sim _0$ indicating the pairs of positions in ${\mathcal {S}}$ that should be merged in a representation reflecting common *k*-mers:

$$\begin{aligned} \langle i,j\rangle \sim _0\langle i',j'\rangle \iff \exists _{0\le m<k}\; S_i[j-m..j+k-1-m]=S_{i'}[j'-m..j'+k-1-m] \end{aligned}$$

Let $\sim$ denote the equivalence closure of $\sim _0$. Obviously, the above definition implies that $S_i[j]=S_{i'}[j']$ whenever $\langle i,j\rangle \sim _0\langle i',j'\rangle$ and, consequently, the same property holds for $\sim$. Therefore a quotient graph *G* of $G_0$ by $\sim$ is properly defined. Moreover, each $G_0$-path $\pi _0(S_i)=\langle v_1,\ldots , v_{|S_i|} \rangle$ can be transformed into *G* through the quotient construction: $\pi (S_i)=\langle [v_1]_{\sim }, \ldots , [v_{|S_i|}]_{\sim } \rangle$.

Hence we have a representation $\langle G,\pi \rangle$ of ${\mathcal {S}}$ that is:

- *k*-complete, because consecutive vertices in paths representing common *k*-mers were merged in the quotient construction,
- *k*-faithful, because all occurrences of a node *v* are in relation $\sim$, so for each pair $\langle i,j\rangle ,\langle i',j'\rangle \in Occ_{\pi }(v)$ there exists a sequence $\langle i,j\rangle =\langle i_0,j_0\rangle ,\ldots ,\langle i_p,j_p\rangle =\langle i',j'\rangle$ of *v*-occurrences such that for each $l\in \{1,\ldots ,p\}$ the condition $\langle i_{l-1},j_{l-1}\rangle \sim _0\langle i_{l},j_{l}\rangle$ is satisfied, which means that occurrences $\langle i_{l-1},j_{l-1}\rangle$ and $\langle i_{l},j_{l}\rangle$ are directly *k*-extendable.

Moreover, by Lemma 1, every representation $\langle G',\pi '\rangle$ of ${\mathcal {S}}$ is isomorphic to a quotient of $G_0$ by some relation $\sim '$ on the set of oriented $G_0$-vertices. It is easily seen that $\langle G',\pi '\rangle$ is

- *k*-complete iff $$\langle i,j\rangle \sim \langle i',j'\rangle \Rightarrow \langle i,j\rangle \sim '\langle i',j'\rangle$$,
- *k*-faithful iff $$\langle i,j\rangle \sim '\langle i',j'\rangle \Rightarrow \langle i,j\rangle \sim \langle i',j'\rangle$$.

Therefore, if $\langle G',\pi '\rangle$ has both properties, graphs *G* and $G'$ must be isomorphic. $\square$

### Bidirected variation graphs

In bidirected graphs each node has two sides (denoted here $\pm 1$) and undirected edges join adjacent nodes on particular sides [18]. Both sides are equivalent in the sense that swapping sides at any node yields an isomorphic bidirected graph.

A path entering a node on one side must exit it on the other side. More formally, a *path* in a bidirected graph is a sequence $\langle \langle v_1,o_1\rangle ,\ldots ,\langle v_m,o_m\rangle \rangle$ such that for all respective *j*

- $o_j=\pm 1$ determines the *orientation* of $v_j$ in the path,
- side $o_{j-1}$ of $v_{j-1}$ is connected by an edge with side $-o_{j}$ of $v_{j}$.

Paths may be reversed, but it requires reversing both order and orientation of the nodes, i.e. given path $p=\langle \langle v_0,o_0\rangle ,\ldots ,\langle v_m,o_m\rangle \rangle$, its reverse is $p^{-1}=\langle \langle v_m,-o_m\rangle ,\ldots ,\langle v_0,-o_0\rangle \rangle$.

*Bidirected variation graphs* naturally represent the double-stranded structure of DNA. The orientation of a node indicates the strand of the represented DNA fragment, i.e. strand $\langle v, +1\rangle$ has sequence *l*(*v*), while $\langle v, -1\rangle$ has sequence $l(v)^{-1}$, where $S^{-1}$ denotes the reverse complement of sequence *S*. For convenience, we introduce notation $S^{+1}=S$ and $p^{+1}=p$. The label of the path is the concatenation of the oriented labels of consecutive vertices, i.e. ${\hat{l}}(\langle \langle v_0,o_0\rangle ,\ldots ,\langle v_m,o_m\rangle \rangle ) = l(v_0)^{o_0}\cdot \ldots \cdot l(v_m)^{o_m}$. Note that hence ${\hat{l}}(p^{-1})={\hat{l}}(p)^{-1}$.

Bidirected representations of sequence collections are defined similarly to directed ones. Positions and vertex occurrences are extended to include the orientation, i.e.

- $$Pos({\mathcal {S}})=\{\langle i,j,o\rangle \;|\; 1\le i\le n \wedge 1\le j\le |S_i|\wedge o=\pm 1\}$$,
- $$Occ_{\pi }(v)=\{\langle i,j,o\rangle \in Pos({\mathcal {S}}) \;|\; \pi (S_i)[j]=\langle v,o\rangle \}$$.

Because the strands of the represented sequences are treated in the same way, the concept of reflecting common *k*-mers applies to occurrences of *k*-mers on both strands. Below we adapt the definitions of *k*-completeness and *k*-faithfulness to take this into account.

Let $S_i,S_{i'}$ be two (not necessarily different) strings from ${\mathcal {S}}$ and assume that they have a common *k*-mer $S_i^{o}[p..p+k-1]=S_{i'}^{o'}[p'..p'+k-1]$ (*o* and $o'$ indicate the strands, on which the *k*-mer occurs). We say that $\pi$ *reflects* this common *k*-mer iff it is represented by the same path in the graph, i.e. $\pi (S_i)^{o}[p..p+k-1]=\pi (S_{i'})^{o'}[p'..p'+k-1]$. We say that $\langle G, \pi \rangle$ represents ${\mathcal {S}}$ *k*-*completely* iff all common *k*-mers in ${\mathcal {S}}$ are reflected by $\pi$.

We say the pair of $\pi$-occurrences $\langle i,j,o\rangle , \langle i',j',o'\rangle$ of a vertex *v* is:

- *directly k-extendable* iff these occurrences extend to a common *k*-mer reflected by $\pi$, i.e. $\pi (S_i)^{o}[j-m..j+m']=\pi (S_{i'})^{o'}[j'-m..j'+m']$ for $m,m'\ge 0$ satisfying $m+m'\ge k-1$,
- *k*-*extendable* if there is a sequence of occurrences of *v* that starts from $\langle i,j,o\rangle$, ends at $\langle i',j',o'\rangle$ and each two consecutive occurrences in that sequence are directly *k*-extendable.

We say that $\langle G, \pi \rangle$ represents ${\mathcal {S}}$ *k*-*faithfully* if every pair of occurrences of a vertex is *k*-extendable.

**Fig. 2:**
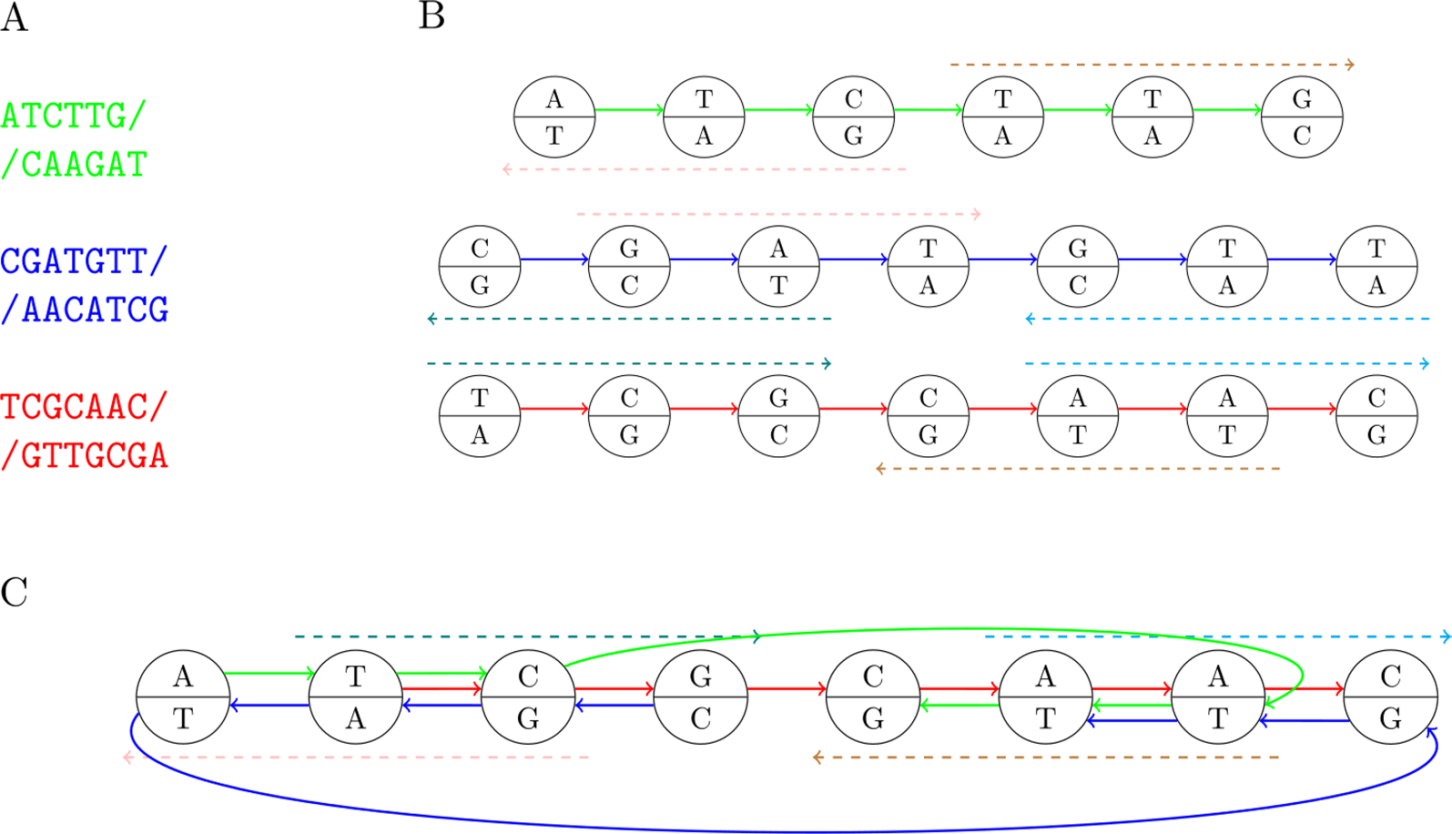
Representing DNA sequences with bidirected variation graphs.** A** Input DNA sequences and their reverse complements.** B** Generic representation of the sequences. Each node is labeled with two complementary nucleotides, from DNA strands $+$ (upper part) and − (lower part). A node has orientation $+1$ on a path that enters it on the left side and exits on the right side, otherwise it has orientation $-1$. Arrows on the edges indicate the orientation of strand $+$ of the represented sequences. The dashed arrows indicate common DNA 3-mers in the input sequences: TTG/CAA, TCG/CGA and AAC/GTT.** C** 3-complete and 3-faithful representation of the sequences. The quotient construction merged each pair of paths in the generic representation indicated by dashed arrows of the same color. Consequently, every 3-mer from the input sequences is represented by a unique path

#### Theorem 2

Let ${\mathcal {S}}=\{S_1,\ldots ,S_n\}$ be a set of DNA sequences. Then the *k*-complete and *k*-faithful representation of ${\mathcal {S}}$ as a singular bidirected variation graph exists and is unique up to isomorphism.

#### Proof

As in the directed case, the desired representation is constructed as a quotient of the generic representation. The definition of the quotient is slightly more complicated in the bidirected case, because each node of the original graph can either retain or reverse its orientation in the resulting graph. In our construction the choice depends on the strands on which the common *k*-mers occur in the ${\mathcal {S}}$-sequences: nodes corresponding to two occurrences of a *k*-mer should have the same orientation when the occurrences lie on the same strand, and the opposite orientation otherwise (see Figure 2). This ensures that the labels of oriented nodes match each other when merging, which means that the quotient is defined correctly. The scheme of the proof and arguments are analogous to the proof of Theorem 1. $\square$

## AlfaPang algorithm

In this section we present AlfaPang – ALignment Free Algorithm for PANGenome graph building. The core part of AlfaPang is based on the quotient construction described in the proofs of Theorems 1 and 2. In subsection "Algorithm overview" we present the algorithm and in subsection "Compact graph representation" we describe an efficient implementation of the data structures it requires. Subsection "Unbranched paths compression" shows how the resulting *k*-complete and *k*-faithful singular variation graph is compressed to a non-singular one. Finally, the computational complexity of AlfaPang is analyzed in the last subsection.

### Algorithm overview

Given a collection of sequences ${\mathcal {S}}$ and a positive natural number *k*, we first build its generic representation $G = \langle V, E \rangle$. Then we build a weighted bipartite graph with parts *V* and *B*, where $V=Pos({\mathcal {S}})$ and *B* is a set of vertices labeled by canonical *k*-mers of ${\mathcal {S}}$, and edges satisfy the following conditions:

- each edge *e* is assigned a value from the set $\{-k, \ldots , -1, 1, \ldots , k\}$, denoted as *C*(*e*). We refer to the absolute value of *C*(*e*) as *color*.
- $$C(\langle \langle i,j\rangle , b\rangle )=c$$ iff
  - $$S_i[j-c+1..j+k-c]=l(b)$$ for $$c>0$$ ,
  - $$S_i[j-c-k..j-c-1]=l(b)^{-1}$$ for $$c<0$$.

Therefore, an edge between $\langle i,j\rangle$ and *b* indicates that the position in the sequence corresponding to $\langle i,j\rangle$ can be extended to a *k*-mer represented by *b*, and the value assigned to the edge indicates its position in that *k*-mer. Hence such graph allows us to represent the relation described in the previous section.

To find all vertices in *G* that should be merged with a chosen vertex *v*, we traverse the bipartite graph starting from *v* using a BFS manner, but with the following constraints:

- If we enter a vertex belonging to *V*, we can leave it by any edge.
- If we visit a vertex belonging to *B* from an edge with color *c*, we can leave it only through edges that share the same color.

**Fig. 3:**
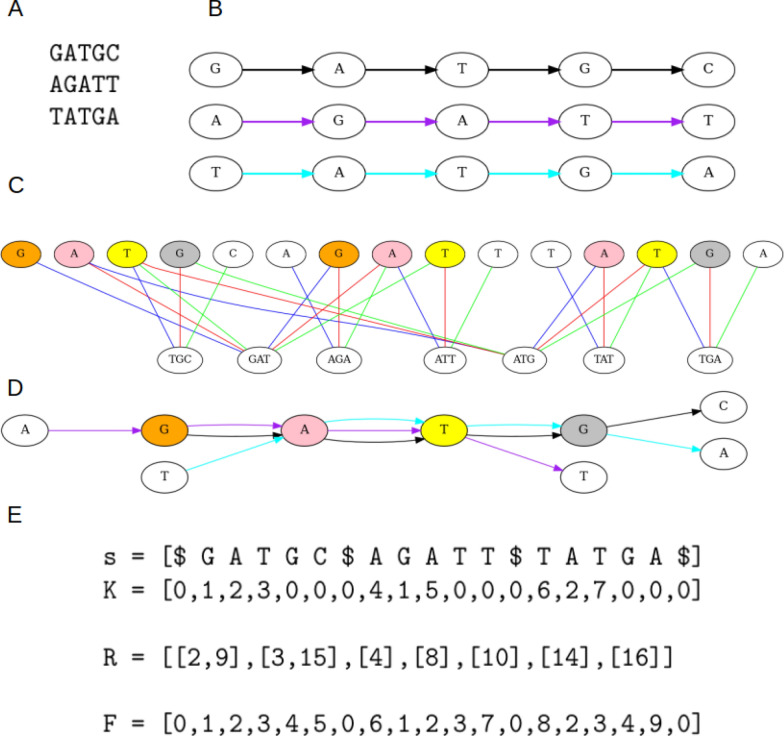
AlfaPang algorithm: construction concept and actual data structures. **A** Input sequences. **B** Generic variation graph representation. **C** Bipartite graph for $k=3$. Colors of edges represent values assigned to them (blue: 1, red: 2, green: 3). Nodes filled with a color other than white belong to the same equivalence class. Orange nodes are both connected to the 3-mer GAT by blue edges. The first and the second pink nodes are connected to GAT by red edges, and the first and third pink nodes are connected to ATG by blue edges. The first and the second yellow nodes are connected to GAT by green edges, and the first and the third are connected to ATG by red edges. Grey nodes are connected to ATG by green edges. **D** Variation graph resulting from the quotient algorithm. Different edge colors mark different genomic paths (not multi-edges) and are consistent with edge colors in B. Node colors are consistent with equivalence classes shown in C. **E** Data structures used in the algorithm: *s* – concatenation of the input sequences, *K* – vector storing ids of *k*-mers starting at given positions in *s*, *R* – inverted index, enabling locating of *k*-mer occurrences in *s*, *F* – output vector, assigning positions in *s* to the vertices of the output graph. To find a pink equivalence class, start from symbol “A” at position 3 (note that we use 1-based indexing) – we assign *F*[3] a new value (2 in this example). Since $K[3] = 2$, we look into the vector *R*[2]. The first entry in this vector is 3, and we used the first color. Therefore we need to visit all other positions pointed to by *R*[2], so we assign $F[15] = 2$ and push it into the queue. Next, we backtrack one position to *K*[2]. Since $K[2] = 1$, we look into *R*[1]. 2 is canonical vertex position for this (*k*-mer, color) pair, so we look at the other elements of *R*[1]. *R*[1] points to position 9, so we move one position forward and assign $F[10] = 2$. After backtracking two steps from the starting position, we find $K[1] = 0$, indicating that this “A” is not the third symbol in any *k*-mer. We repeat the procedure from found positions 10 and 15, identifying no additional positions

All vertices of *V* visited during one such run establish one equivalence class of the relation presented in the theorems. For each such class, we choose a canonical orientation arbitrarily to be consistent with a canonical label of the first vertex visited in the run. Therefore, to find a quotient graph, we start a new run as long as there are vertices not visited in previous runs. The simplified (directed graph-based) version of the above construction is illustrated on Figure 3A-D.

### Compact graph representation

We reduce the memory requirements of our algorithm by representing the redundant information from the bipartite graph implicitly. First, we store only the edges with values 1 or $-k$ and calculate the rest on the fly. This optimization is based on the observation that if $e_1 = \{\langle i, j \rangle , b\}$ and $C(e_1) = 1$, then for $1< q < k$ and $e_2 = \{\langle i, j-q \rangle , b\}$, we have $C(e_2) = 1 + q$. Similarly, if $e_1 = \{\langle i, j \rangle , b\}$ and $C(e_1) = -k$, then for $1< q < k$ and $e_2 = \{\langle i, j-q \rangle , b\}$, we have $C(e_2) = -k + q$. We can then modify the constraints for graph traversal:

- From a node belonging to an equivalence class, we can exit by an edge or jump $q < k$ positions backward in the generic representation and then traverse through the edge incident with that vertex (this vertex is not marked as visited since it does not need to belong to the same class).
- From vertices belonging to *B*, we can exit by both types of edges (those assigned to 1 and those assigned to $-k$).
- If we exit from a node belonging to *B* by an edge with the same sign as the edge we used to enter that vertex, we need to jump *q* vertices forward in the generic representation to find a vertex belonging to the equivalence class.
- If we exit from a vertex belonging to *B* by an edge with the opposite sign to the one we used to enter that vertex, we need to jump $k - 1 - q$ vertices forward to find a vertex belonging to the equivalence class.

To implement the algorithm, we do not build such a graph explicitly. Instead, we use the following data structures (see example on Figure 3E):

- A concatenation of all sequences from the set $${\mathcal {S}}$$ as a single string *s*, with an additional special character $ at the ends of original sequences.
- A vector *K* of the same size as *s*, that stores the ID (positive integer) assigned to the *k*-mer *b* at position *i* if $$s[i..i+k-1]=b$$, or the negative of that value if $$S[i..i+k-1]=b^{-1}$$. If the *k*-mer starting at position *i* is not fully included in a sequence from *S*, then $$K[i]=0$$. IDs of all canonical *k*-mers of $${\mathcal {S}}$$ are obtained by enumeration with positive numbers, which can be done efficiently using a hash table. Our implementation uses a hash table from an external library unordered_dense [19] and the hashing algorithm ntHash [20], designed specifically for DNA sequences.
- A vector *R* that stores *M* vectors, where *M* is the number of distinct canonical *k*-mers in $${\mathcal {S}}$$. This vector is used as an inverted index, enabling fast locating of *k*-mer occurrences in *s*. If for some $$i,j > 0$$ we have $$K[i] = \pm j$$, then we push *i* into the vector *R*[*j*].
- A vector *F* of the same size as *s*, initially filled with zeros. This vector represents the assignment of positions in *s* to the vertices of the output graph. The absolute value of a number in this vector identifies a vertex ID, and its sign determines the vertex orientation.
- Moreover, we retain the order in which the sequences of $${\mathcal {S}}$$ were concatenated, allowing us to track different genomes and reconstruct genomic paths from *F*.

In such framework we can identify vertices belonging to *V* with indices of *K* and vertices belonging to *B* with indices of *R*.

To traverse the graph under the assumed constraints, we should store in a queue the visited *V*-nodes and pairs $\langle B$-node, edge color$\rangle$. Due to the number of possible pairs, this can lead to excessive memory complexity. To avoid this, for each pair $\langle b,c\rangle$ we define its *canonical* *V*-*node* – the node adjacent to *b* via an edge of color *c* with the lowest *K*-index. We update the graph traversal condition as follows: when traversing an edge from a *V*-node to a *B*-node, we check whether the *V*-node is canonical for the *B*-node and the edge color. If it is, we add to queue all other vertices adjacent via edges of the same color from the *B*-node. Otherwise, we enqueue only the canonical *V*-node. In both cases, the nodes are added to the queue unless they have already been visited, which is checked in the vector *F*.

The pseudocode of the algorithm is presented in Algorithm 1.

**Algorithm 1:**
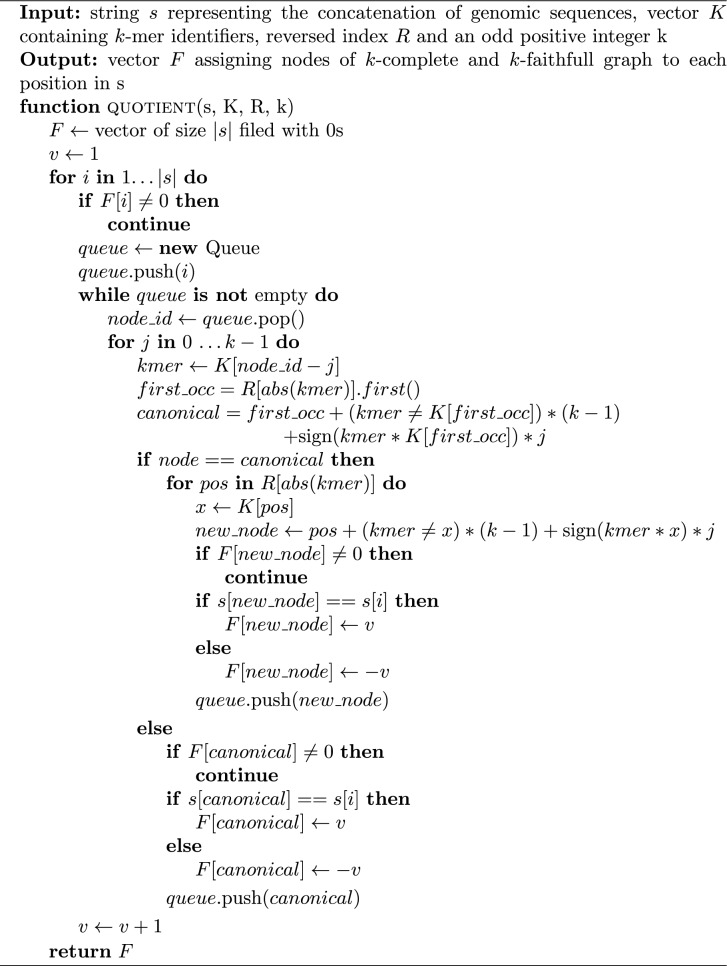
Quotient Algorithm

### Unbranched paths compression

The graph resulting from the described algorithm is singular. As a final step in our tool, we compress unbranched paths into vertices to reduce its size. Since it is k-complete, we know that, (similarly to the vertices of the de Bruijn graph) each vertex has no more than $|\Sigma |$ vertices connected to it on each side, and each of these has a different symbol as a label. Therefore, based on ideas proposed in [21], we implemented a similar algorithm for our variation graphs. For each vertex, we can assign a state that encodes whether the vertex is inside such a path or is a branching node. Initially, vertices are assigned to one of the $(\Sigma +1)^2$ states representing symbols on their sides (+1 for a special character representing the end of a sequence), or to one of three special states representing branching vertices - two different states for situations when a vertex has only one edge on one side and more than one edge on the other, and a state for vertices with more than one edge on each side. To recognize branching vertices, we build a vector *D* of length equal to the number of vertices in the singular graph, filled with 0. Then we traverse vector *F* and update *D* as follows:

- If $D[abs(F[i])]=0$, we assign it a state encoding labels of the previous and next vertex ($s[i-1]$ and $s[i+1]$).
- Otherwise, we check if labels of vertices on both sides of a vertex are consistent with the state assigned to it. If so, we do nothing. Otherwise, we change the state to one of the special states (depending on which side of the vertex differs).
- If a vertex is assigned to a special state, we need to check only one of the sides or none of them.
- Additionally, we check if $-F[i-1] = F[i]$ or $F[i]=-F[i+1]$. If so, we also need to change the state to one of the specials.

Next, we simplify states by changing all non-special states to a single value. Then, we traverse vector *F* once again to merge maximal unbranching paths into vertices, identified by combinations of branching nodes at the ends of the paths.

### Algorithm complexity

Below we analyze the complexity of the subsequent steps of AlfaPang. In all the following lemmas *N* denotes the total size of the collection of genomic sequences ${\mathcal {S}}$, *k* is the chosen *k*-mer size and *M* is the number of distinct *k*-mers in ${\mathcal {S}}$. We start by examining the construction of the input structures for the Quotient Algorithm: *s*, *K*, and *R* (see Figure 3).

#### Lemma 2

All input structures for the Quotient Algorithm can be build from ${\mathcal {S}}$ in ${\mathcal {O}}(kN)$ time and ${\mathcal {O}}(N)$ space.

#### Proof

Each data structure used by the Quotient Algorithm has a total size of ${\mathcal {O}}(N)$.

To construct the vector *K*, we iterate over the string *s* exactly once, using an additional hash table to assign identifiers to *k*-mers. Since we can use pointers to the first occurrence of each *k*-mer in *s* as keys (instead of storing the *k*-mers explicitly), size of the table is ${\mathcal {O}}(M)$. However, key comparisons require ${\mathcal {O}}(k)$ time, so the overall time complexity for constructing *K* is ${\mathcal {O}}(kN)$.

To construct the reverse index *R*, we initialize a vector of length *M*, where each entry is an empty vector. We then traverse *K* once, filling values in *R*, which requires ${\mathcal {O}}(N)$ operations. $\square$

In the next lemma we investigate the complexity of the Quotient Algorithm.

#### Lemma 3

The Quotient Algorithm runs in ${\mathcal {O}}(kN)$ time and uses ${\mathcal {O}}(N)$ space.

#### Proof

Since the Quotient Algorithm consists of traversing all edges of a bipartite graph – essentially performing a breadth-first search with additional constraints on the edges used – its time complexity is ${\mathcal {O}}(kN)$.

The only additional structures used during execution are the output vector *F*, which is of size the same length as *s*, and a FIFO queue storing nodes to be processed. The modified procedure of passing through the *B*-nodes enables us to store only *V*-nodes, so the total size of the queue is bounded by *N*. $\square$

Finally, we examine the complexity of the output graph compression.

#### Lemma 4

The unbranched paths are compressed in ${\mathcal {O}}(N)$ time and space.

#### Proof

For unbranched path compression, we construct a state vector *D* with the number of elements equal to the number of vertices in the singular graph, which is bounded by *N*. We then traverse the vector *F*, obtained from the Quotient Algorithm, once to assign values in *D*. Next, we traverse *D* again to change all non-special values to zero.

Branching vertices are identified in *F* as those assigned nonzero values in *D*. Each compressed vertex is uniquely defined by its endpoints, so we initialize a hash table that assigns consecutive integers to such pairs. We also initialize vectors for paths in the compressed graph and a hash table linking each compressed vertex identifier to its label, derived from the indices of its first occurrence.

Finally, a single traversal of *F* populates these structures, all of which have size ${\mathcal {O}}(N)$ and require ${\mathcal {O}}(N)$ time. $\square$

The following theorem summarizes the above results.

#### Theorem 3

The AlfaPang algorithm runs in ${\mathcal {O}}(kN)$ time and uses ${\mathcal {O}}(N)$ space.

#### Proof

Follows directly from Lemmas 2-4. $\square$

## Design of experiments

### Datasets and *k*-mer length selection

We tested our tool on two series of genome collections: the *Escherichia coli* series containing 50, 100, 200, and 400 *Escherichia coli* haplotypes, obtained from [13], which we further extended by datasets of 800, 1600 and 3412 sequences downloaded from GenBank (with total lengths ranging from 250*Mbp* to 17.3*Gbp*; specific queries are available in our GitHub repository), and the *Saccharomyces cerevisiae* series containing 16, 32, 64, and 118 haplotypes (total length from 195*Mbp* to 1.44*Gbp*), obtained from [22].

Before conducting the analysis, we assessed the complexity and repetitiveness of the sequences in the datasets to guide the choice of the parameter *k* for AlfaPang. Too small *k* may lead to merging non-homologous fragments, while too large *k* may hinder the detection of sequence similarity.

To explore this, we selected one dataset with an intermediate number of genomes from each series (*E. coli* 100 and *S. cerevisiae* 64) and calculated the fraction of *rare* *k*-mers, i.e. occurring only once in the entire dataset. We used KMC3 [23] to compute this fraction across a selected range of *k*. Since the fraction of rare *k*-mers increases approximately linearly with *k*, we estimated it at a few key points. For both datasets, we examined graph structures for values of *k* yielding rare *k*-mer fractions of 0.1, 0.2, 0.3, 0.5, and 0.10. For each case, we measured the maximum *node coverage* (the number of occurrences of the node in all genomic paths) and the fraction of the pangenome located in *private* nodes (having coverage equal to 1). The results are summarized in Table 1.

**Table 1:** Graph properties for selected values of *k* corresponding to specific rare *k*-mer fractions

rare *k*-mers fraction	0.01	0.02	0.03	0.05	0.10
*E. coli *100					
*k*	15	47	83	165	379
Fraction of sequence in private nodes	0.003	0.009	0.012	0.020	0.040
Max node coverage	244604	768	685	574	470
*S. cerevisiae* 64					
*k*	11	27	45	77	163
Fraction of sequence in private nodes	0.000	0.003	0.005	0.009	0.020
Max node coverage	$2.7\times 10^8$	10894	6839	5116	3498

Based on these observations, we decided to use a *k* yielding 5% rare *k*-mers for all AlfaPang tests. We assumed that this choice would maintain the fraction of private nodes at approximately 1-2% while ensuring a reasonable maximum node coverage. This assumption was confirmed in tests on all graphs. The selected values for each dataset are summarized in Table 2.

Although the above approach provides practical advice on how to choose *k*, the final decision is up to the user. It may depend not only on the properties of the dataset being analyzed (number of genomes, their length, repetitiveness, etc.), but also on the level of separation of input genomes required in the subsequent analysis.

**Table 2:** Values of *k* chosen for different datasets

Sequences	*E. coli*							*S. cerevisiae*			
	50	100	200	400	800	1600	3412	16	32	64	118
*k*	47	165	217	267	371	533	745	47	47	77	117

### Compared algorithms

In the pggb pipeline, the preliminary graph constructed in its two first steps (wfmash+seqwish) is further locally refined in the third step. Similarly to seqwish, AlfaPang may produce pangenome graphs with complex local structures due to the close relationship to de Bruijn graphs. We therefore evaluate AlfaPang on two levels.

On the first level, we compared the computational efficiency of AlfaPang and the first two steps of the pggb workflow, i.e. wfmash+seqwish. Because we were unable to complete the wfmash+seqwish calculations even for the 400 *E. coli* sequences (after 6 days of computations on a server with 512 GB RAM, the process crashed due to excessive memory consumption), we repeated the calculations on the *E. coli* series with the Erdős-Rényi random graph sparsification option activated. With that approach, we computed variation graphs for 400 and 800 sequences, but 1600 still required too much memory.

In order to prepare the second-level comparison, we modified the pggb pipeline by replacing the first two steps with AlfaPang. Thus, the new workflow, called AlfaPang+, consists of AlfaPang followed by the graph refinement tools smoothxg and gfaffix. For all *S. cerevisiae* datasets and datasets of 50, 100, 200, and 400 *E. coli* sequences, we compared AlfaPang+ with pggb and Minigraph-Cactus in terms of computational efficiency and output graph properties.

In the case of *S. cerevisiae* datasets we applied all tools in two ways: to the entire genomes and to each of 16 yeast chromosomes separately (after removing from genomes aneuploid sequences). Both approaches are practiced in pangenome analysis. Moreover, Minigraph-Cactus remaps all genomic sequences onto the initial graph and assigns each sequence to one of the reference chromosomes. Sequences that cannot be confidently assigned to any chromosome are left out of further analysis. Thus, subsequent steps may be performed independently and in parallel for each reference chromosome, resulting in significant computational speedup for multichromosomal genomes. This comes at the cost of leaving interchromosomal homologies not included in the graph. Our experiments allow us to assess the importance of this factor for the results of comparing the performance of the three tools.

## Results

### Performance comparison

The performance of each tool was evaluated in terms of running time and peak memory consumption. Running time was measured as wall clock time, and peak memory as maximum resident set size using the |time| command for all tests, except for parallel AlfaPang runs on *S. cerevisiae* chromosomes, where |cgmemtime| was used for peak memory measurement. All benchmarks were performed on a Supermicro X10DRi server with 512GB RAM and two 14-cores CPUs Intel Xeon E5-2690V4, using single thread for AlfaPang applied to entire genomes, 16 threads for AlfaPang applied to each chromosome separately, and 20 threads for all other tools.

**Fig. 4:**
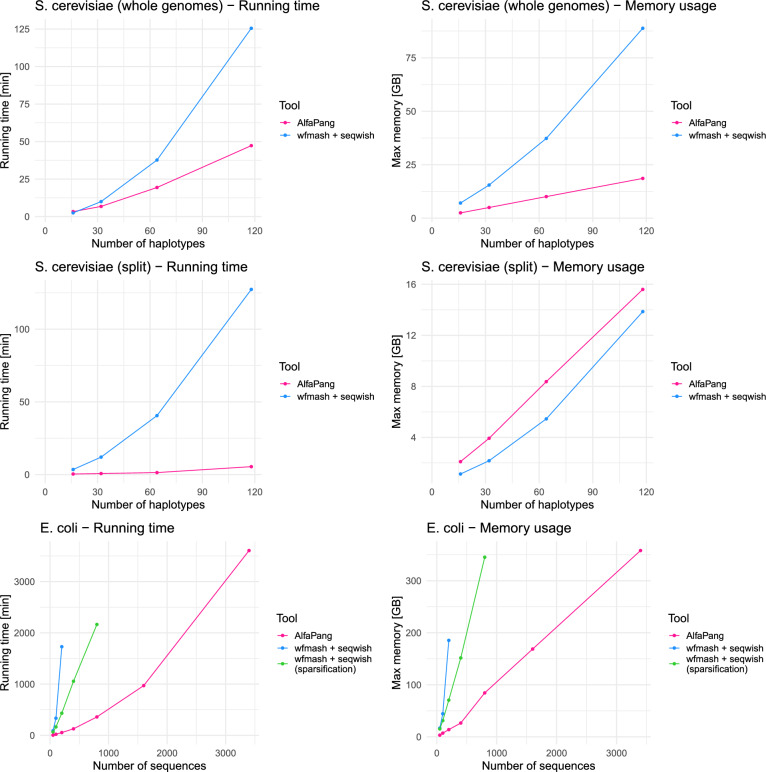
Plots of performance of AlfaPang vs wfmash+seqwish. All wfmash+seqwish tests were performed using 20 CPU threads. For graph construction with splitting by chromosome 16 AlfaPang processes were running in parallel

Figure 4 presents the comparison of AlfaPang and wfmash+seqwish, without the refinement step of pggb. For construction from whole genomes AlfaPang consumes less memory than wfmash+seqwish on all datasets and is substantially faster than it on all the datasets except the smallest *S. cerevisiae* dataset. Moreover, unlike wfmash+seqwish, AlfaPang scales almost linearly with respect to the number of genomes. The deviation from perfect linearity is due to the dependence of the time complexity on the parameter *k*. This is most visible in the *E. coli* dataset series, where the *k* values chosen for the largest and smallest datasets differ by more than 15 times. Additionally, AlfaPang has a memory usage increase when scaling from 400 to 800 *E. coli* genomes due to the need to switch from 4-byte to 8-byte integers, but linear scaling resumes afterward. Despite the above deviations from linearity, the difference between AlfaPang and wfmash+seqwish grows with the dataset size. For example, on 100 *E. coli* sequences, AlfaPang is more than 20 times faster than wfmash+seqwish (and 15 times faster than wfmash+seqwish with activated sparsification) and consumes 5 times less memory, while on *E. coli* 200, the largest dataset where a full wfmash+seqwish run was successfully completed, AlfaPang is more than 30 times faster and consumes 13 times less memory.

It is worth pointing out that AlfaPang achieved these results using only one thread, while wfmash+seqwish was tested on 20 CPU threads. For the construction of a separate graph for each *S. cerevisiae* chromosome, we run 16 AlfaPang processes in parallel. This allowed us to outperform the wfmash+seqwish pipeline on all datasets, although at the cost of higher memory usage, as wfmash+seqwish processed each chromosome sequentially using 20 CPUs, while AlfaPang+ processed all 16 chromosomes simultaneously.

**Fig. 5:**
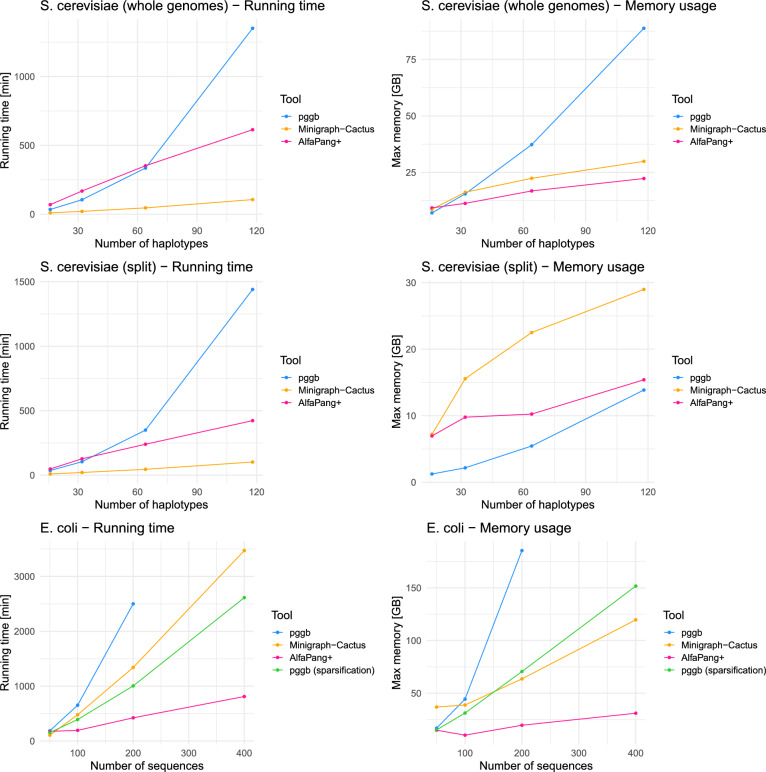
Performance of AlfaPang+ vs pggb vs Minigraph-Cactus. All pggb and Minigraph-Cactus tests, as well as smoothxg in AlfaPang+ were performed using 20 CPU threads. For graph construction with splitting by chromosome 16 AlfaPang processes were running in parallel

Figure 5 summarizes the computational efficiency of full pangenome building pipelines. For the series of *E. coli* datasets, AlfaPang+ proved to be the most efficient tool and shows almost linear scalability with respect to the size of the data.

On the dataset consisting of 400 *E. coli* sequences, AlfaPang+ is more than 3 times as fast as pggb (with sparsification enabled) and 4 times faster than Minigraph-Cactus, while using 4-5 times less memory than other tools.

For the series of *S. cerevisiae* datasets, the results are slightly more ambiguous. As the number of *S. cerevisiae* haplotypes goes from 16 to 118, the memory usage of algorithms applied to whole genomes increases by 12.5 times for pggb, 3.4 times for Minigraph-Cactus, and 2.4 times for AlfaPang+, which again demonstrates AlfaPang+ scalability in this regard. The runtime for both pggb and AlfaPang+ is mostly dependent on smoothxg, which runs faster in pggb. However, as the number of haplotypes increases, AlfaPang+ gains an advantage and becomes twice as fast for the largest dataset. Minigraph-Cactus proved to be the fastest tool in this case. For construction from whole genomes it is 3 to 13 times faster than pggb, depending on the number of haplotypes, and 6-8 times faster than AlfaPang+. This comes at the cost of loosing interchromosomal structural variants in the resulting variation graph. As opposite to pggb and AlfaPang+, for all yeast datasets Minigraph-Cactus constructed graphs with 16 connected components, corresponding to 16 chromosomes of *S. cerevisiae*.

When applying the tools separately to each chromosome, Minigraph-Cactus is still the fastest, but the difference in running time between AlfaPang+ and Minigraph-Cactus is visibly reduced (pggb takes almost the same amount of time as without splitting). Furthermore, both pggb and AlfaPang+ outperforms Minigraph-Cactus in terms of memory usage.

### Graphs topology

To measure the complexity of the produced pangenome graphs, we compared such graph characteristics as the number of nodes and edges. Results are displayed in Tables 3 and 4. We also compared the total length of nodes labels to check the ability of all the tools to compress input sequences (see Table 5). In all the following tables, we refer to the graphs produced by the pggb pipeline without sparsification, except for the graph of the largest *E. coli* dataset, for which sparsification was activated.

**Table 3:** Number of nodes (in $10^6$)

Dataset	AlfaPang+	pggb	*Minigraph-cactus*
*S. cerevisiae* 16	0.92	0.89	1.08
*S. cerevisiae* 32	1.78	1.80	2.22
*S. cerevisiae* 64	2.75	2.85	3.86
*S. cerevisiae* 118	3.61	3.87	5.44
*S. cerevisiae* 16*	1.00	0.90	1.08
*S. cerevisiae* 32*	1.91	1.79	2.21
*S. cerevisiae* 64*	2.78	2.83	3.78
*S. cerevisiae* 118*	3.65	3.85	5.22
*E. coli* 50	1.74	1.62	1.93
*E. coli* 100	1.76	1.79	2.67
*E. coli* 200	2.49	2.61	4.13
*E. coli* 400	3.27	3.25	5.80

* Graphs constructed separately for each chromosome

**Table 4:** Number of edges (in $10^6$)

Dataset	AlfaPang+	pggb	*Minigraph-Cactus*
*S. cerevisiae* 16	1.27	1.22	1.48
*S. cerevisiae* 32	2.47	2.48	3.05
*S. cerevisiae* 64	3.87	3.98	5.37
*S. cerevisiae* 118	5.11	5.49	7.68
*S. cerevisiae* 16*	1.37	1.23	1.47
*S. cerevisiae* 32*	2.63	2.46	3.03
*S. cerevisiae* 64*	3.85	3.93	5.26
*S. cerevisiae* 118*	5.08	5.43	7.37
*E. coli* 50	2.37	2.20	2.64
*E. coli* 100	2.40	2.45	3.65
*E. coli* 200	3.42	3.60	5.72
*E. coli* 400	4.52	4.53	8.16

* Graphs constructed separately for each chromosome

**Table 5:** Total number of base pairs in nodes (in $10^6$ bp)

Dataset	AlfaPang+	pggb	*Minigraph-Cactus*	Input size
*S. cerevisiae* 16	12.61	17.03	18.59	192.04
*S. cerevisiae* 32	13.64	20.73	26.54	384.52
*S. cerevisiae* 64	16.01	26.52	38.58	769.61
*S. cerevisiae* 118	19.37	29.56	53.67	1416.23
*S. cerevisiae* 16*	14.10	16.78	17.95	189.02
*S. cerevisiae* 32*	15.93	20.68	24.71	374.53
*S. cerevisiae* 64*	20.33	26.25	34.12	746.54
*S. cerevisiae* 118*	26.28	30.89	47.69	1376.10
*E. coli* 50	14.02	19.01	28.89	249.52
*E. coli* 100	17.92	20.20	43.61	539.83
*E. coli* 200	28.59	27.37	67.28	1050.67
*E. coli* 400	43.37	68.21	90.75	2027.21

* Graphs constructed separately for each chromosome

For *E. coli* and *S. cerevisiae* datasets constructed for entire genomes, the number of nodes in graphs from AlfaPang+ and pggb differs by at most 7%. For graphs constructed separately for each chromosome, the differences are up to 10%. Minigraph-Cactus produces graphs with a noticeably larger number of nodes and edges. On *S. cerevisiae* datasets, Minigraph-Cactus graphs contain 17-50% and 8-43% more nodes than AlfaPang+ for graphs without and with chromosome splitting, respectively (20-40% more than pggb). On *E. coli* data, Minigraph-Cactus graphs have 10-77% more nodes than AlfaPang+ (19-78% more than pggb).

For all graphs, the number of edges is 35-45% larger than the number of nodes. Moreover, going from 16 to 118 *S. cerevisiae* haplotypes increases the number of nodes 4-5 times, while going from 50 to 400 *E. coli* sequences, the number of nodes increases by at most 3 times across all tools, indicating that for all tools graph size grows sublinearly with respect to the number of input sequences. Similar conclusion can be drawn from Table 5. When the number of *S. cerevisiae* haplotypes increases from 16 to 118, the total size of node sequences increases by 1.5, 1.7, and 2.9 times for AlfaPang+, pggb, and Minigraph-Cactus, respectively. For *E. coli* data, increasing the number of sequences from 50 to 400 results in the total size of node sequences increasing by around 3 times for both AlfaPang+ and Minigraph-Cactus. For pggb, the graph constructed from a dataset of 400 sequences deviates from this trend: increasing the number of sequences from 50 to 200 increases the total length of node labels by 1.4 times, while increasing from 200 to 400 sequences increases this value by 2.5 times. This deviation is suspected to result from the sparsification used for that dataset. For all datasets, AlfaPang+ shows a higher rate of compression than pggb, which in turn has a higher rate of compression than Minigraph-Cactus.

### Graphs similarity

Below we analyze the similarity between sequence alignments induced by the graphs constructed with different tools. To define the measure of this similarity, we introduce the following equivalence relation: a pair of positions in two (not necessary different) sequences is *aligned* in graph *G* if both are represented by the same position in a label of the same vertex. In the case of singular graphs this could be expressed $S_i[p] \equiv _{G} S_j[p'] \iff \pi (S_i)[p]=\pi (S_j)[p']$. We define $\mu (G)$ as the set of all aligned pairs in *G*. The similarity between two variation graphs $G_1, G_2$ is measured using Jaccard index between $\mu (G_1)$ and $\mu (G_2)$.

Table 6 summarizes the number of aligned pairs in all graphs. With some exceptions, AlfaPang+ found more pairs than pggb. For whole-genome *S. cerevisiae* series, pggb identified 69-84% as many pairs as AlfaPang+. For the split-chromosome series, these values are much higher and pggb found 96-99% as many pairs as AlfaPang+, except for the largest dataset, in which case pggb identified more pairs than AlfaPang+. For *E. coli* datasets, pggb found 91-97% as many pairs as AlfaPang+ (except for the dataset with 200 sequences, in which case pggb found approximately 4% more pairs than AlfaPang+), while Minigraph-Cactus found 73-85%.

As expected, the number of aligned pairs scales quadratically with the number of sequences.

**Table 6:** Number of aligned positions pairs (in $10^9$)

Dataset	*AlfaPang+*	pggb	*Minigraph-Cactus*
*S. cerevisiae* 16	2.20	1.52	1.26
*S. cerevisiae* 32	8.47	6.33	5.14
*S. cerevisiae* 64	33.81	25.81	21.00
*S. cerevisiae* 118	108.69	90.95	71.50
*S. cerevisiae* 16*	1.45	1.40	1.25
*S. cerevisiae* 32*	5.79	5.65	4.99
*S. cerevisiae* 64*	22.64	22.32	20.06
*S. cerevisiae* 118*	76.61	76.67	68.94
*E. coli* 50	5.55	5.08	4.72
*E. coli* 100	26.09	25.31	19.04
*E. coli* 200	88.94	92.37	73.70
*E. coli* 400	354.57	343.97	298.79

* Graphs constructed separately for each chromosome. A pair of positions from input sequences is aligned in a graph if they are both represented by the same position in a label of the same vertex

**Table 7:** The values of the Jaccard index between sets of aligned pairs

Dataset	pggb vs AlfaPang+	Minigraph-Cactus vs AlfaPang+	Minigraph-Cactus vs pggb
*S. cerevisiae* 16	68.3	56.8	81.6
*S. cerevisiae* 32	72.9	59.9	80.0
*S. cerevisiae* 64	72.9	60.9	79.9
*S. cerevisiae* 118	76.5	64.4	77.2
*S. cerevisiae* 16*	95.3	85.4	88.4
*S. cerevisiae* 32*	95.8	85.1	87.2
*S. cerevisiae* 64*	95.1	86.6	88.4
*S. cerevisiae* 118*	94.6	87.1	88.4
*E. coli* 50	88.5	82.7	91.4
*E. coli* 100	84.3	69.8	74.1
*E. coli* 200	83.0	73.7	78.2
*E. coli* 400	77.4	70.5	84.5

* Graphs constructed separately for each chromosome

**Fig. 6:**
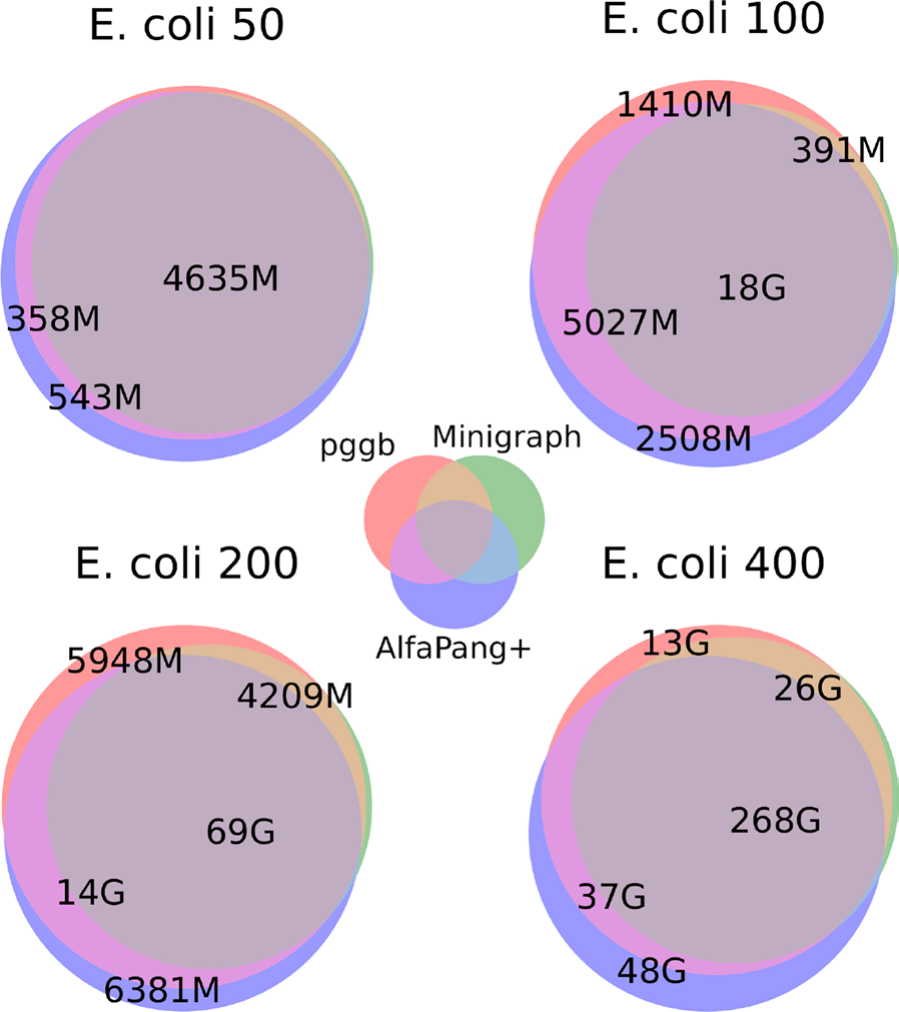
Venn diagrams for sets of aligned pairs in graphs build with pggb, Minigraph-Cactus and AlfaPang+ for *E. coli* datasets

**Fig. 7:**
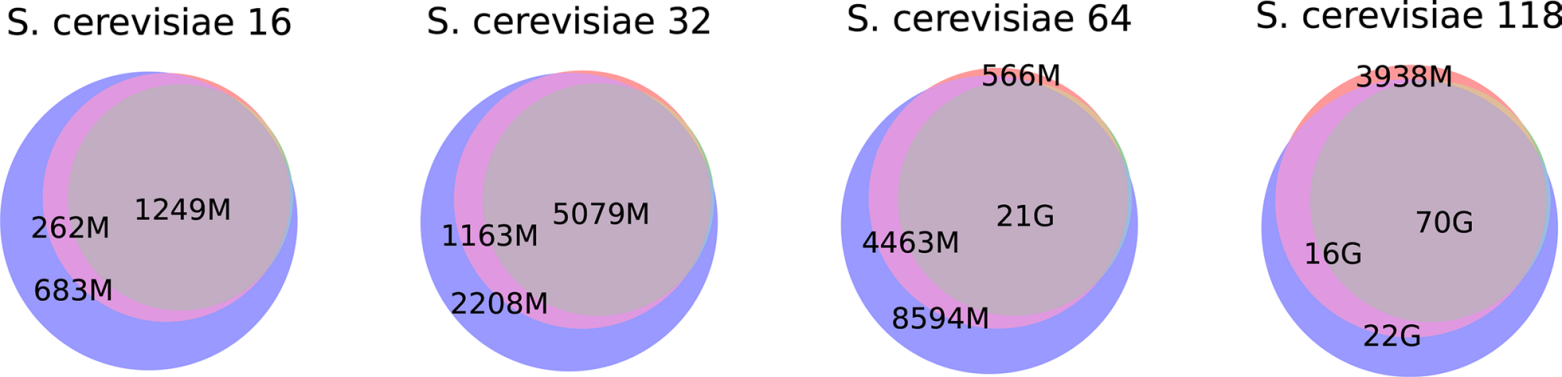
Venn diagrams for sets of aligned pairs in graphs build with pggb, Minigraph-Cactus and AlfaPang+ for whole *S. cerevisiae* datasets. Subsets are colored as in Figure 6

**Fig. 8:**
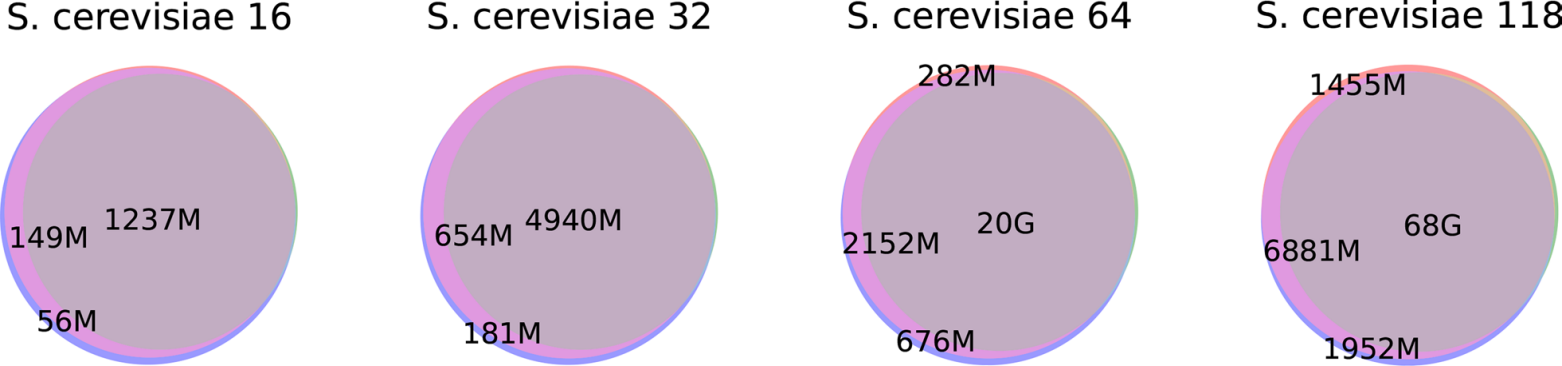
Venn diagrams for sets of aligned pairs in graphs build with pggb, Minigraph-Cactus and AlfaPang+ for *S. cerevisiae* datasets split by chromosomes. Subsets are colored as in Figure 6

The Jaccard index between sets of aligned pairs is presented in Table 7. On the *S. cerevisiae* datasets constructed for whole genomes, the Jaccard index between AlfaPang+ and pggb increases from 68% to 77% as the number of haplotypes grows, while for the pair AlfaPang+ and Minigraph-Cactus an increase from 56% to 64% is observed. The Jaccard index for pggb and Minigraph-Cactus ranges between $82\%$ and $87\%$ on *S. cerevisiae* datasets, with a decreasing tendency as the number of haplotypes grows. For graphs constructed separately for each chromosome, these values are noticeably higher: approximately $95\%$ for AlfaPang+ and pggb, $85-87\%$ for AlfaPang+ and Minigraph-Cactus, and $87-88\%$ for pggb and Minigraph-Cactus.

For *E. coli* datasets, the Jaccard index between AlfaPang+ and pggb and between AlfaPang+ and Minigraph-Cactus decreases as the number of sequences increases. For the first mentioned pair, the index drops from $89\%$ to $77\%$, and for the second one from $83\%$ to $70\%$. In contrast, the Jaccard index between pggb and Minigraph-Cactus varies between 74 and $91\%$, showing no clear dependencies on the number of sequences.

The values of the Jaccard index are very close to the ratios of the numbers of aligned pairs between the tools. A more detailed analysis of these relationships is presented in Figures 6, 7 and 8. It shows that, in *E. coli* datasets, AlfaPang+ is able to identify 88–99% of the pairs found by both pggb and Minigraph-Cactus. The proportion of AlfaPang+ pairs found by pggb varies between 86% and 93% with no clear dependencies on the number of sequences (for Minigraph-Cactus, it varies between 71% and 84%).

For *S. cerevisiae* datasets without splitting, AlfaPang+ is able to find 95–99% of the pairs identified by pggb and 98–99% of those found by Minigraph-Cactus. In comparison, pggb and Minigraph-Cactus find 68–80% and 57–65% of the pairs identified by AlfaPang+, respectively. Similarity is much higher when the graphs are constructed separately for each chromosome. Namely, AlfaPang+ identifies over 97% and 98% of pggb and Minigraph-Cactus pairs, respectively, while pggb finds over 95% of AlfaPang+ pairs and Minigraph-Cactus finds 85–88% of AlfaPang+ pairs. For all datasets, the set of aligned pairs found by Minigraph-Cactus is almost entirely included in set found by pggb.

Thus, we conclude that the differences between pair sets are mainly due to the differences in sensitivity to sequence similarity between tools.

### Aligned pairs annotations

Below we investigate whether differences among pangenome graphs produced by various tools occur in functionally important regions. To do this, we first extract aligned pairs from each graph and randomly sample 1,000,000 pairs per graph. Next, we classify each genomic position based on overlapping features using GenBank annotations, considering positions without any feature as intergenic. Finally, we analyze the distribution of annotation pairs. Since classifications other than gene, pseudogene (only in E. coli), or intergenic are exceedingly rare, our analysis focuses exclusively on these categories.

**Fig. 9:**
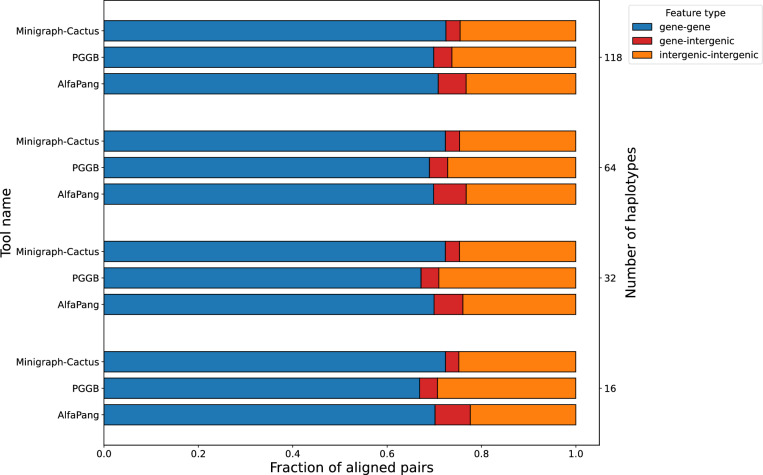
Distribution of aligned pair classes in graphs constructed with AlfaPang+, pggb, or Minigraph-Cactus for *S. cerevisiae* datasets (whole genomes). The notion of an aligned pair is defined in Section "Graphs similarity"

**Fig. 10:**
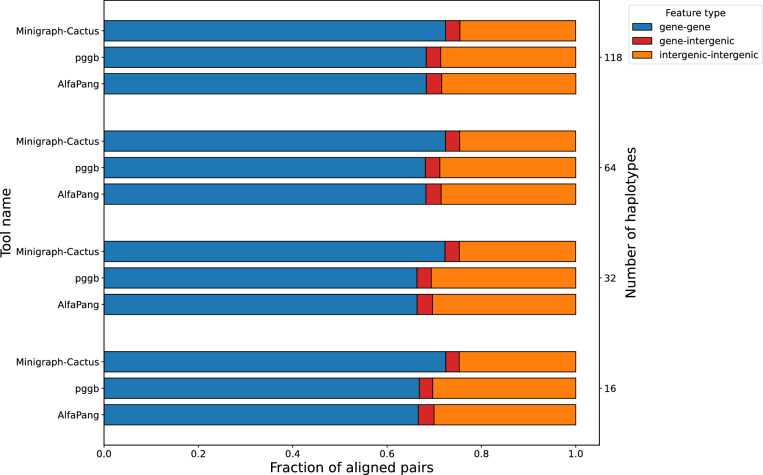
Distribution of aligned pair classes in graphs constructed with AlfaPang+, pggb, or Minigraph-Cactus for *S. cerevisiae* datasets (spliting). The notion of an aligned pair is defined in Section "Graphs similarity"

**Fig. 11:**
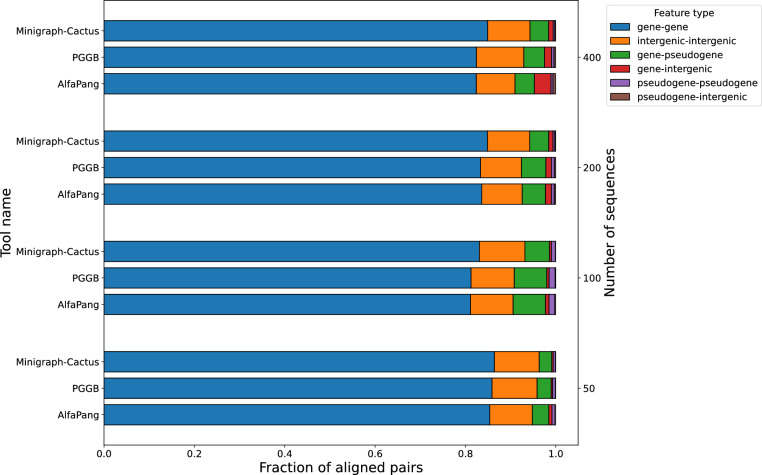
Distribution of aligned pair classes in graphs constructed with AlfaPang+, pggb, or Minigraph-Cactus for *E. coli* datasets. The notion of an aligned pair is defined in Section "Graphs similarity"

In Figure 9 we show the annotation distributions on *S. cerevisiae* data without splitting. Approximately 70-71% of AlfaPang+ pairs were located in genes, 22–24% were classified as intergenic-intergenic, and 6–7% as gene-intergenic. For pggb, these values are 67–69%, 27–29%, and 3–4% and for Minigraph-Cactus it was 72%, 25% and 3% respectively.

Annotation distributions for *S. cerevisiae* data divided into chromosomes are presented in Figure 10. These values are very close to each other for both AlfaPang+ and pggb, with 66–68% gene-gene pairs, 28–30% intergenic-intergenic pairs, and around 3% gene-intergenic pairs. Minigraph-Cactus shows a slightly higher proportion of gene-gene pairs (68–72%) and a lower proportion of intergenic-intergenic pairs (23–24%).

Figure 11 presents annotation distributions for *E. coli* genomes. For datasets with 50 and 100 and 200 sequences, the proportion of gene-gene pairs was similar across all tools (85%, 86%, and 86% for 50 sequences, 81%, 81%, and 83% for 100 sequences and 84%, 83% and 85% for 200 sequences for AlfaPang+, pggb, and Minigraph-Cactus, respectively). However, for AlfaPang+, the proportion of gene-intergenic pairs was slightly higher, at 0.7% compared to 0.3% in both pggb and Minigraph-Cactus for 50 sequences, and 0.7% compared to 0.5% and 0.4% in pggb and Minigraph-Cactus for 100 sequences and 1.3% compared to 1.2% and 1% for 200 sequences.

For 400 sequences, the proportion of gene-gene pairs in AlfaPang+ graph remains similar to the other tools - 82% compared to 82% in pggb and 85% in Minigraph-Cactus. On the other hand, the proportion of gene-intergenic pairs was significantly higher in AlfaPang+, at 3.7%, compared to 1.5% in pggb and 1% Minigraph-Cactus.

## Conclusion

We presented AlfaPang – a novel algorithm for building pangenome graphs. Unlike alternative algorithms, AlfaPang constructs graphs with their structure strictly defined by the *k*-completeness and *k*-faithfulness properties introduced in [17]. The runtime and memory usage of AlfaPang scales linearly with the number of genomes, allowing it to process much larger sets of genomes than state-of-the-art alternatives such as wfmash+seqwish. Replacing the latter with AlfaPang in the pggb pipeline results in output graphs with similar properties in terms of graph structure, but with a larger number of aligned genome residues. Although the decision whether or not given fragments of genomic sequences should be aligned in a pangenome graph is somewhat arbitrary, this fact reflects the high sensitivity of AlfaPang to sequence similarity.

Another state-of-the-art pangenome graph builder tool, Minigraph-Cactus, substantially differs from both pggb and AlfaPang in the assumptions on how the pangenome graph should look like. First, it aligns neither paralogs (i.e. similar sequence fragments in the same genome) nor homologuous sequences that in different genomes occur on different chromosomes. Second, it requires the user to choose a reference genome. This choice highly influences the output graph, as it may ignore similarity between fragments of non-reference genomes that have no homologs in the reference. Both design assumptions make it possible to reduce the number of sequence alignments necessary to build the graph, which consequently allows Minigraph-Cactus to provide much better computational efficiency than pggb. However, the alignment-free approach of AlfaPang allows even greater reduction, especially in terms of required memory, and this difference increases with the size of the dataset.

Current population sequencing projects aim to assemble the genomic sequences of thousands of individuals. The success of pangenome-based approaches in analyzing the resulting genome collections depends largely on the existence of efficient methods for building pangenomes. Because the total size of such data is in the terabytes, memory efficiency may be of paramount importance here, and it may turn out that the computational advantages of AlfaPang will enable new insights and discoveries that are not possible using other tools.

The close relationship between the variation graphs constructed by AlfaPang and the de Bruijn graphs provides a bridge between both pangenome models. On the other hand, this is a limitation of the AlfaPang approach, as the structure of resulting graphs resembles the structure of de Bruijn graphs with their drawbacks, such as excessive entanglement in areas representing low-complexity sequence regions. Such entanglement is removed in the refinement step of the AlfaPang+ pipeline by the smoothxg tool. However, due to high AlfaPang efficiency, this step dominates the whole AlfaPang+ computation time ($\sim 95\%$ on all datasets). Perhaps more precise tuning of the smoothxg parameters would allow to reduce this time without affecting the output. More substantial reduction would probably require incorporation of the refinement procedure in the graph building process of AlfaPang.

## Data Availability

C++ implementation of AlfaPang can be found at: https://github.com/AdamCicherski/AlfaPang.
